# Prescribing errors in post - COVID-19 patients: prevalence, severity, and risk factors in patients visiting a post - COVID-19 outpatient clinic

**DOI:** 10.1186/s12873-022-00588-7

**Published:** 2022-03-05

**Authors:** Rashudy F. Mahomedradja, Tessa O. van den Beukel, Maaike van den Bos, Steven Wang, Kirsten A. Kalverda, Birgit I. Lissenberg-Witte, Marianne A. Kuijvenhoven, Esther J. Nossent, Majon Muller, Kim C. E. Sigaloff, Jelle Tichelaar, Michiel A. van Agtmael

**Affiliations:** 1 Department of Internal Medicine, Section Pharmacotherapy, Amsterdam UMC location VUmc, Amsterdam, The Netherlands; 2 Department of Internal Medicine, Research and Expertise Center in Pharmacotherapy Education (RECIPE), Amsterdam UMC location VUmc, Amsterdam, The Netherlands; 3 Department of Internal Medicine, Geriatric Medicine Section, Amsterdam Cardiovascular Sciences Institute, Amsterdam UMC, location VUmc, Amsterdam, The Netherlands; 4 Department of Pulmonary Medicine, Amsterdam UMC location AMC and location VUmc, Amsterdam, The Netherlands; 5 Department of Epidemiology and Data Science, Amsterdam UMC location VUmc, Vrije Universiteit Amsterdam, Amsterdam, The Netherlands; 6 Department of Clinical Pharmacology and Pharmacy, Amsterdam UMC location VUmc, Amsterdam, The Netherlands; 7 Amsterdam UMC, Vrije Universiteit Amsterdam, Department of Internal Medicine, Division of Infectious Diseases, Amsterdam Institute for Infection and Immunity, De Boelelaan 1117, Amsterdam, The Netherlands

**Keywords:** COVID-19, Prescribing errors, Pharmacotherapeutic stewardship, Clinical pharmacology

## Abstract

**Background:**

Severe acute respiratory syndrome coronavirus 2 (SARS-CoV-2), which causes coronavirus disease 2019 (COVID-19), has challenged healthcare globally. An acute increase in the number of hospitalized patients has necessitated a rigorous reorganization of hospital care, thereby creating circumstances that previously have been identified as facilitating prescribing errors (PEs), e.g. a demanding work environment, a high turnover of doctors, and prescribing beyond expertise. Hospitalized COVID-19 patients may be at risk of PEs, potentially resulting in patient harm. We determined the prevalence, severity, and risk factors for PEs in post–COVID-19 patients, hospitalized during the first wave of COVID-19 in the Netherlands, 3 months after discharge.

**Methods:**

This prospective observational cohort study recruited patients who visited a post-COVID-19 outpatient clinic of an academic hospital in the Netherlands, 3 months after COVID-19 hospitalization, between June 1 and October 1 2020. All patients with appointments were eligible for inclusion. The prevalence and severity of PEs were assessed in a multidisciplinary consensus meeting. Odds ratios (ORs) were calculated by univariate and multivariate analysis to identify independent risk factors for PEs.

**Results:**

Ninety-eight patients were included, of whom 92% had ≥1 PE and 8% experienced medication-related harm requiring an immediate change in medication therapy to prevent detoriation. Overall, 68% of all identified PEs were made during or after the COVID-19 related hospitalization. Multivariate analyses identified ICU admission (OR 6.08, 95% CI 2.16–17.09) and a medical history of COPD / asthma (OR 5.36, 95% CI 1.34–21.5) as independent risk factors for PEs.

**Conclusions:**

PEs occurred frequently during the SARS-CoV-2 pandemic. Patients admitted to an ICU during COVID-19 hospitalization or who had a medical history of COPD / asthma were at risk of PEs. These risk factors can be used to identify high-risk patients and to implement targeted interventions. Awareness of prescribing safely is crucial to prevent harm in this new patient population.

**Supplementary Information:**

The online version contains supplementary material available at 10.1186/s12873-022-00588-7.

## Key messages

- What is the key question?

What are the prevalence, severity, and risk factors for prescribing errors in post - COVID-19 patients?

- What is the bottom line?

Prescribing errors occurred frequently during the SARS-CoV-2 pandemic. These errors can lead to adverse drug events, resulting in medication-related harm and even hospital (re)admissions.

Identified risk factors for prescribing errors are ICU admission and a medical history of COPD / asthma. These risk factors should be used to identify high-risk patients and to develop targeted interventions.

- Why read on?

Risk factors for prescribing errors identified in a non-pandemic situation are not always relevant in a pandemic. We provide an overview of how the changing circumstances in a pandemic influence in-hospital prescribing, and what the consequences are for patients and medication safety.

## Introduction

Severe acute respiratory syndrome coronavirus 2 (SARS-CoV-2), which causes coronavirus disease 2019 (COVID-19), has challenged healthcare globally. In the Netherlands, the first case was confirmed on February 27, 2020 and thereafter the original Wuhan Hu-1 strain of SARS-CoV-2 spread rapidly throughout the country [1, 2]. This led to a sudden, sharp increase in the number of patients in acute need of hospitalization [3]. In order to manage, hospital services were reorganized – regular care was scaled down, clinical wards were separated into non-, suspected, and proven COVID-19 units, and intensive care unit (ICU) capacity was increased. Healthcare professionals from all medical specialties and levels of experience joined the frontline to provide COVID-19 care [4]. The novelty of COVID-19 meant that it was not clear how to treat the disease [5–7]. Intense efforts to learn about the pathophysiology of COVID-19 [8] resulted in the use and subsequent disuse of various medical treatments [9–11] and to rapidly changing guidelines on disease management.

Prescribing medication beyond the prescriber’s expertise, insufficient prescribing skills, a demanding work environment, rapidly changing guidelines, a high turnover of patients and doctors, and multiple transfers of care are associated with prescribing errors (PEs) [12–17], leading to adverse drug events (ADEs) [18]) [19] and potentially medication-related harm and hospital (re)admission [20]. In times of scarce hospital capacity and resources, such as during a pandemic, this can put extra pressure on already overstretched hospital services. We hypothesized that hospitalized COVID-19 patients were at risk of PEs, potentially resulting in medication-related harm requiring additional care. We therefore determined the prevalence, severity, and risk factors for PEs in COVID-19 patients 3 months after they had been discharged from hospital during the first wave of SARS-CoV-2 in the Netherlands, when they attended a post COVID-19 outpatient clinic (PCOC). Such information obtained during a pandemic can be used to develop management strategies to cope with new waves of COVID-19 or other pandemics [21].

## Methods

### Study design and setting

This prospective observational single center cohort study was performed following the Strengthening the Reporting of Observational Studies in Epidemiology (STROBE) statement (Table S1). Longitudinal analysis was performed to evaluate pharmacotherapeutic care during the first wave of SARS-CoV-2 in Amsterdam UMC - location VUmc, a 733-bed academic hospital in the Netherlands accredited by the Joint Commission International. The hospital has an active, multidisciplinary Medication Committee, consisting of medical safety officers from various backgrounds and a multidisciplinary pharmacotherapy team [12, 22], that monitors medication safety in daily in-hospital practice.

The Medical Ethics Review Board of the Amsterdam UMC – location VUmc approved the study procedures (no. 2021.0090).

### Participants & general PCOC procedures

Patients were considered eligible if they were hospitalized in Amsterdam UMC, location VUmc, between March 1 and July 1 2020 for i) COVID-19 or ii) other reasons and developed COVID-19 during hospitalization and were scheduled for a PCOC appointment between June 1 and October 12,020.

**Fig. 1:**
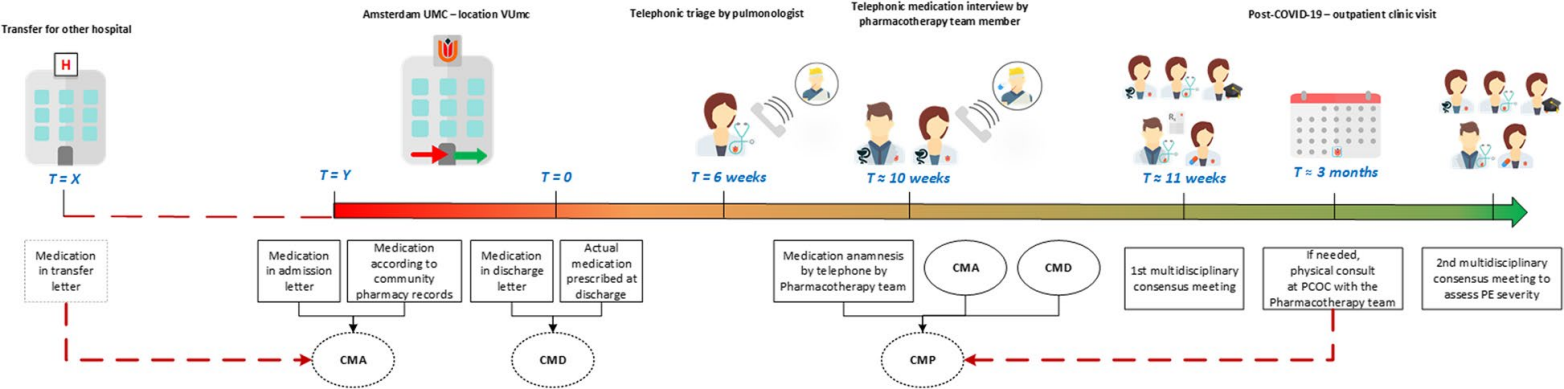
Flowchart of patient journey of COVID-19 patients at Amsterdam UMC and the intervention of the Pharmacotherapy team. Six weeks after hospital discharge (T = 6 weeks), telephonic triage by a pulmonologist took place to determine if follow up was necessary at the post – COVID-19 outpatient clinic of Amsterdam UMC location VUmc (PCOC), planned approximately 3 months after hospital discharge (T ≈ 3 months). CMA: consensus medication list at admission at Amsterdam UMC location VUmc. In case patient was transferred from another hospital, the information from the transfer letter was included in the CMA; CMD: consensus medication list at discharge from Amsterdam UMC location VUmc (T = 0); CMP: consensus medication list at PCOC (T = approximately 3 months after hospital discharge)

Approximately 6 weeks after discharge, all post – COVID-19 patients were contacted by telephone by a pulmonologist and invited to come to the PCOC if they had not fully recovered, if their SARS-CoV-2 infection had required ICU admission, or if they had had a venous thromboembolic event (VTE). All PCOC appointments were scheduled approximately 3 months after discharge (Fig. 1).

Before the PCOC appointment, patients had a full blood screen, pulmonary function tests, an electrocardiogram (ECG), and a chest computed tomography (CT) scan, and were asked to fill out questionnaires that asked questions about physical functioning, nutrition, and cognition. The outcomes of these questionnaires were outside the scope of this study. During the PCOC visit, patients were assessed by a physical therapist and a pulmonologist. A multidisciplinary pharmacotherapeutic stewardship team (MP-team) reviewed each patient’s medication use. The MP-team consisted of a junior medical doctor and junior pharmacist, supported by a medical student and supervised by an internist and a hospital pharmacist some of whom were training to be clinical pharmacologists.

### Data collection

#### Definitions

A PE was defined based on the definition of Dean et al. [23] and included two subtypes: inappropriate prescriptions and unintentional drug discrepancies [24] (Table 1). Unintentional drug discrepancies were subsequently categorized in 4 subcategories, covering ‘unintentional initiation of a drug’, ‘unintentional omission of a drug’, ‘unintentional switch of a drug within the same Anatomical Therapeutic Chemical (ATC) group’, and ‘unintentional change of a drug dosage’ (Table 1).

**Table 1:** Definitions

Term	Definition
1. Adverse drug events (ADE) [18]	“An injury resulting from medial intervention related to drug”
2. Prescribing error (PE) [23]	“An error in prescribing decision(s) and/or the (electronic) prescription writing process that could result in clinically relevant and significant harm to the patient or to a diminished effect of treatment”
3. Unintentional drug discrepancy [24]	A change in prescribed medication, without there being a documented rationale for this change.
3.1. Unintentional initiation of a drug	Medication was initiated i) even though there is no rationale or indication documented justifying initiation; ii) even though there is a rationale documented where a drug was discontinued and should stay discontinued but was re-initiated.
3.2. Unintentional omission of a drug	Medication was not prescribed and there was no documented rationale justifying discontinuation.
3.3. Unintentional switch of a drug within the same Anatomical Therapeutic Chemical (ATC) group	Medication was switched to another drug within the same ATC group, even though there was no rationale documented justifying the switch.
3.4. Unintentional change of a drug dosage	Dosage was changed either to a higher or lower dosage even though there was no rationale documented justifying the change.
4. Inappropriate prescription	Deviations in medication therapy as stated in hospital-, national- (e.g. The Royal Dutch Pharmacists Association database), or international evidence-based guidelines. In case there were pathophysiological and/or evidence-based reasons for deviations from these evidence-based guidelines recorded in the patient’s medical record, this was not considered inappropriate by the pharmacotherapy team.
4.1. ‘Underuse’	- Incomplete pharmacotherapy according to relevant guideline or protocol; - Incorrect duration (too short) of prescribed drug therapy.
4.2. ‘Overuse’	- Drug continued despite no indication (anymore) (desprescribing); - (Pseudo) drug duplication.
4.3. Potentially inappropriate medications	Drug should be discontinued due to an adverse drug event (ADE), in toleration or contra-indication
4.4. Incorrect dosing	- Under- or overdosing - Incorrect dosing frequency
5. Medication reconciliation [25]	“The process of obtaining and maintaining a complete and accurate list of the patients’ current medication use across healthcare settings” performed by trained pharmacy technicians

Inappropriate prescriptions were determined based on adherence to local-, national- (e.g. The Royal Dutch Pharmacists Association database (‘KNMP Kennisbank’.)), or international evidence-based guidelines. COVID-19 medication management was assessed according to local and national guidelines. Deviations from these evidence-based guidelines with pathophysiological and/or evidence-based arguments recorded in the patient’s medical record were not considered as inappropriate. Inappropriate prescriptions were categorized based on the core outcome set for appropriate medication use of Spinewine et al. [26], including ‘medication overuse’, ‘medication underuse’, ‘potentially inappropriate medications’, and ‘clinically significant drug-drug interactions’ (Table 1).

#### Procedure

Up to 2 weeks before the scheduled PCOC appointment, information on patient characteristics and medication use (both at hospital admission and hospital discharge) was collected by the MP-team, using a standard form (Table S2). Subsequently, eligible patients were contacted by telephone for a medication interview.

If the medication interview and subsequent assessment could not be completed, patients were scheduled for a face-to-face consultation with a member of the MP-team during their PCOC visit. Patients were excluded from analysis if they did not have a medication interview or if they did not show up for their PCOC appointment. One week before the patient’s scheduled PCOC appointment, a multidisciplinary consensus meeting, attended by members of the MP-team plus a rotating fellow in clinical pharmacy, was held to discuss the data collected and to establish a consensus medication list at admission (CMA), a consensus medication list at discharge (CMD), and a consensus medication list at time of the PCOC visit (Fig. 1), based on information from the patient’s community pharmacist’s records, the admission letter, transfer letter (if applicable), discharge letter to the general practitioner, and the medication interview. PEs were identified by determining the presence of inappropriate prescriptions at the time of the PCOC visit, and unintentional drug discrepancies introduced between hospital admission and hospital discharge, and between hospital discharge and the PCOC visit.

Any identified PEs and subsequent suggestions for medication optimization were recorded in the electronic patient record and communicated to the pulmonologists of the PCOC. The pulmonologist decided whether these suggestions were to be implemented directly during the patient’s PCOC visit or subsequently by the patient’s general practitioner (via a letter sent after patient’s PCOC visit), or whether they were to be rejected.

After a patient’s PCOC visit, a second consensus meeting was held, attended by all members of the MP-team, to assess 1) the moment when the PE was introduced (before, during or after COVID-19 – related hospitalization); 2) if the PE resulted in patient harm according to the European Medicine Agency (EMA) classification tool [27] (Fig. S1), and, if applicable, the severity of the PE, according to the index of the National Coordinating Council for Medication Error Reporting and Prevention (NCC MERP) [28], specifically NCC MERP categories E - I (Table S3). This assessment was done based on all data collected by the MP-team, the pulmonologist, and physical therapist during the PCOC visit. All data were recorded in a password-protected electronic case report form (eCRF) (Castor EDC).

### Outcomes

The primary outcomes were the total number of PEs identified at the time of the PCOC visit. Secondary outcomes were the severity, risk factors for, and the number of PEs made during and after COVID-19-related hospitalization.

### Data analysis and statistical methods

Variables are described in terms of frequencies and percentages for categorical variables and median values (interquartile range (IQR) and range) for non-normally distributed continuous variables.

Risk factors for the presence of ≥1 PE at the time of the PCOC visit were identified by univariate analysis. The association between dichotomous risk factors and each of the outcome measures was investigated with the chi-square test or Fisher’s exact test (in case of < 10 patients with at least one PE). Logistic regression models were used to investigate the association between continuous risk factors and each outcome measure. After the identification of individual risk factors associated with PEs, a multivariable logistic regression model was built, using a forward selection procedure, to identify independent risk factors for each outcome measure. Risk factors with a *p*-value < 0.1 in the univariate analyses were preselected for the multivariable model. The *p*-value for inclusion in the final multivariable model was set at 0.05. All analyses were carried out with SPSS 26 for Windows statistical software.

### Study size

Owing to the limited number of patients scheduled for a PCOC appointment, a post-hoc power analysis based on a power of 80%, a type 1 error (α) of 5%, was performed to test the reliability of multivariable analysis.

## Results

Between July 1 and October 12,020, 102 patients had a PCOC appointment. Four patients were excluded from analysis: two patients were not available for the medication interview, and did not come to their appointment and two patients were not sufficiently fluent in Dutch for a reliable medication interview, such that a face-to-face consultation was necessary, but the patients did not come to their appointment. Ninety-eight patients were included in this analysis (Table 2).

**Table 2:** Patient characteristics

		Total included patients (*n* = 98)
**Demographics at hospital admission**		
Age in years	Median (IQR^a^)	61.0 (50.5–70.3)
	Range	18.0–86.0
Gender	Male (%)	67.3
BMI in kg/m^2^	Median (IQR^a^)	27.3 (24.6–30.1) (*N* = 87)
	Range	18.8–40.0
Smoker	Yes (%)	6.1 (*N* = 80)
Number of patients with no (blanc) medical history	%	28.6
Charlson Comorbidity Index	Median (IQR^a^)	2 (1–3)
	Range	1–6
Charlson Comorbidity Index 0	%	20.4
Charlson Comorbidity Index 1	%	17.3
Charlson Comorbidity Index 2	%	21.4
Charlson Comorbidity Index 3	%	22.4
Charlson Comorbidity Index 4	%	13.3
Charlson Comorbidity Index 5	%	3.1
Charlson Comorbidity Index 6	%	2.0
Living situation prior to COVID-19 hospitalization:		
at home without professional care	%	88.8
at home with professional care	%	5.1
at home with informal care	%	3.1
at a nursing home	%	1.0
homeless	%	1.0
Transferred from another hospital	Yes (%)	27.6
Number of prescriptions at admission according to CMA	Median (IQR^a^)	3.0 (1.0–6.0)
	Range	0–16
Number of drugs in admission letter of hospital of first COVID-19 presentation^c^	Median (IQR^a^)	2.0 (1.0–5.0)
	Range	0–15
**Hospitalization characteristics**		
Regular medication reconciliation performed at hospital admission	Yes (%)	7.1
ICU admission during hospitalization	Yes (%)	36.7
Intubated at ICU	Yes (%)	80.6 (*N* = 36)
Received renal replacement therapy at ICU	Yes (%)	11.1 (*N* = 36)
Total duration of ICU admission in days	Median (IQR^a^)	12.0 (6.25–18.75) (*N* = 36)
	Range	1.0–61.0
Complications during hospitalization	Yes (%)	44.9
Pulmonary embolism	%	18.4
Delirium that required treatment with medication	%	22.4
Infection other than SARS-CoV-2 infection	%	11.2
Deep vein thromboembolism	%	3.1
Myocardial infarction	%	2.0
Atrial fibrillation	%	4.1
Ischemic or hemorrhage stroke	%	5.1
Pericarditis	%	4.1
Number of intramural transfers^b^ (i.e. transfers between departments within the Amsterdam UMC - location VUmc	Median (IQR^a^)	1 (0–1)
	Range	0–8
Duration of hospitalization in days	Median (IQR^a^)	8.5 (4.0–19.0)
	Range	1–70
**After hospital discharge**		
Living situation direct after discharge:		
home without professional care	%	56.1
home with professional care	%	3.1
home with informal care	%	3.1
Temporary rehabilitation center ultimately to home	%	32.7
a nursing home	%	4.1
homeless	%	1.0
Number of prescriptions at discharge according to CMD	Median (IQR^a^)	5.0 (3.0–9.0)
	Range	0–15
Number of drugs in discharge letter^d^	Median (IQR^a^)	5.0 (3.0–9.0)
	Range	0–17
**At time of PCOC**		
Living situation at time of PCOC visit:		
home without professional care	%	84.7
home with professional care	%	5.1
home with informal care	%	5.1
Temporary rehabilitation center ultimately to home		2.0
a nursing home	%	1.0
homeless	%	1.0
unknown	%	1.0
Number of prescriptions in CMP	Median (IQR^a^)	3.5 (1.0–7.0)
	Range	0–19
Number of OTC drugs	Median (IQR^a^)	0 (0–1.0)
	Range	0–8

^a^ Interquartile range with lower and upper quartile

^b^ the number of transfers between departments/wards within Amsterdam UMC - location VUmc

^c^ Either of Amsterdam UMC – location VUmc or of another hospital

^d^ of Amsterdam UMC – location VUmc

### Participant characteristics

At admission, the median age was 61 (IQR 50.5–70.3; range 18–86) years, 67% were male, the median BMI was 27.3 (IQR 24.6–30.1), and the median Charlson Comorbidity Index was 2 (IQR 1–3; range 0–6). Twenty-seven patients (28%) had been transferred from another hospital to Amsterdam UMC – location VUmc. Medication reconciliation was performed for 7 patients (7%) at hospital admission. The median number of prescriptions according to the consensus medication list at admission was 3.0 (IQR 1.0–6.0). Most patients (*n* = 87; 89%) lived at home without professional care prior to hospitalization (Table 2).

Included patients had been hospitalized for a median of 8.5 days (range 1.0–70.0 days). Of these patients, 36 (37%) had been admitted to the ICU during hospitalization, for a e median of 12 days (range 1–61 days). Reported complications during COVID-19 hospitalization were mainly delirium (22%), pulmonary embolism (18%), or an infection other than SARS-CoV-19 (11%) (Table 2).

The median number of prescriptions according to the consensus medication list at discharge was 5.0 (IQR 3.0–9.0). At discharge, 55 (56%) patients returned home without professional care, whereas 32 patients (33%) were discharged temporarily to a rehabilitation center (Table 2).

The median (range) time between hospital discharge and the PCOC visit was 120.5 (61.0–210.0) days. The median number of prescribed drugs in the consensus medication list at time of the PCOC visit was 3.5 (IQR 1.0–7.0; range 0–19) and the number of over-the-counter (OTC) drugs used according to patients was 0 (IQR 0–1.0; range 0–8). At time of the PCOC visit, 84 patients (86%) lived at home without professional care (Table 2).

### Prevalence, severity, and risk factors for prescribing errors and when these errors were made

In total, 139 PEs, affecting 90 patients (92%), were identified at the time of the PCOC visit – 67 inappropriate medications (48%; *N* = 139) and 72 unintentional drug discrepancies (52%) (Table 3). The drugs most-often associated with a PE were those for acid-related disorders (ATC code A02) due to ‘overuse’, meaning prescribed or maintained without an appropriate medical indication (Table S5).

**Table 3:** Prevalence and severity of prescribing errors identified from pharmacotherapeutic assessment

Type of Prescribing Errors (PEs) [22]		Absolute number of PEs introduced during hospitalization	Absolute number of PEs introduced after discharge	Total number of PEs	Total number of patients experiencing patient harm according to the EMA classification ‡
**Unintentional discrepancies** [23]		between CMA* and CMD**	between CMD** and CMP***		
**Unintentional initiation of a drug**		30	4	**34**	5
**Unintentional omission of a drug**		18	9	**27**	3
**Unintentional switch of a drug within the same ATC-group**		1	2	**3**	Δ
**Unintentional dosage change**		5	3	**8**	Δ
**Total number of unintentional discrepancies**		**54**	**18**	**72 (100%)**	**Δ**
**Inappropriate medication use**					
**Core outcome** [24]	**Specification**				
**Underuse**	Incomplete pharmacotherapy according to relevant guideline or protocol	0	6	**6**	1
	Incorrect duration (too short) of prescribed drug therapy	0	1	**1**	Δ
**Overuse**	Drug continued despite no indication (anymore) (*desprescribing*)	3	3	**6**	Δ
	(Pseudo) drug duplication	1	0	**1**	Δ
	No, unknown or incorrect indication of a drug	0	1	**1**	Δ
**–**	Incorrect dosing *- Under- or overdosing* *- Incorrect dosing frequency*	3	3	**6**	1
**Potentially inappropriate medications**	Drug should be discontinued due to an adverse drug event (ADE), intoleration or contraindication	2	0	**2**	2
**Total number of inappropriate medications**		**9 (13.4%)**	**14 (20.9%)**	**67 (100%) †**	**Δ**
**Total number of PEs**		**63 (45.3%)**	**32 (23.0%)**	**139 (100%) †**	**Δ**

* Consensus medication list at admission

** Consensus medication list at discharge

*** Consensus medication list at post – COVID-19 outpatient clinic

Δ Not applicable

◊ Prescribing error; an unintentional discrepancy or an error in inappropriate prescription

† including 44 inappropriate medications introduced prior to COVID-19 – hospitalization at Amsterdam UMC – location VUmc

‡ It could occur ≥1 PE resulting in patient harm was identified in one unique patient

Sixteen PEs (12%; *N* = 139), affecting eight patients (8%), resulted in patient harm according to the EMA classification. Three patients had more than one PE resulting in harm. The severity of these 16 PEs was categorized as NCC MERP E, meaning that the PEs may have contributed to temporary patient harm and required an immediate change in medication therapy to prevent deterioration [28] (Table S3). Examples of PEs and their severity are presented in Table S4. None of the PEs resulting in patient harm involved drugs prescribed for acid-related disorders (ATC code A02) or vitamins (ATC code A11) (Table S5).

Most (45%; *N* = 139) of the identified PEs occurred between hospital admission and discharge; 23% occurred between hospital discharge and the PCOC visit. Thus 32% of the identified PEs, all categorized as inappropriate medications, were present before hospitalization (Table 3).

Of the PEs made between hospital admission and discharge, 11 (8%) caused harm in five patients (5%) (Table 3); 4 of the PEs made between hospital discharge and the PCOC visit caused harm in three patients (3%). Thus only 1 PE that caused patient harm was made before COVID-19-related hospitalization.

**Fig. 2:**
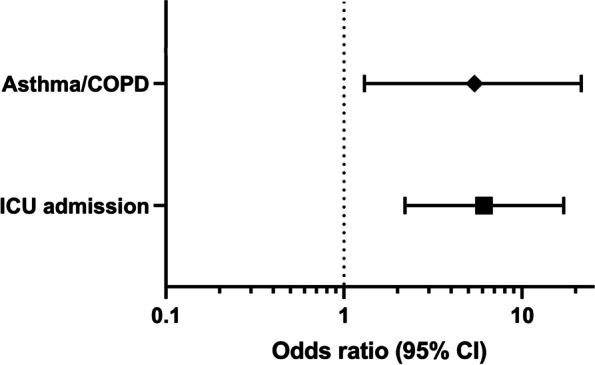
Results of multivariate analysis determining risk factors associated with prescribing errors (PEs) during the course of COVID-19 hospitalization

Univariate analysis identified risk factors associated with PEs made between hospital admission and discharge and/or between hospital discharge and PCOC visit (Table S6): ICU admission during hospitalization (*p* < 0.001), a medical history of COPD or asthma (*p* = 0.005), and a medical history of hypertension (*p* < 0.001). Multivariate analysis demonstrated that ICU admission during COVID-19 hospitalization and a medical history of COPD / asthma were risk factors for the identified PEs (odds ratio (OR) 6.08, 95% CI, 2.16–17.09, and OR 5.36, 95% CI, 1.34–21.5, respectively) (Fig. 2; Table S6). Our sample achieves 91% to detect an OR of 6.08 for ICU admission and 65% power to detect and OR of 5.36 for a medical history of COPD / asthma as risk factors for potentially harmful PEs, suggesting that findings were reliable.

## Discussion

In this study, we evaluated medication safety during the first wave of the SARS-CoV-2 pandemic by determining the prevalence, severity, and risk factors for PEs detected approximately 3 months after COVID-19-related hospitalization. PEs were found in the medication lists of most of the patients (92%) who came to the PCOC. Sixteen PEs that caused patient harm were identified in the medication lists of eight patients and necessitated a change in therapy. We hypothesized that hospitalized COVID-19 patients were at risk of PEs due to the novelty of the disease and rapidly changing management guidelines [5–7]. Study of the pathophysiology of COVID-19 [8] led to the rise and fall of medication therapies to treat COVID-19, for example remdesivir [9, 29], (hydroxy)chloroquine in combination with azitromycin [10, 30], ritonavir combined with lopinavir (Kaletra®) [31] and corticosteroids [8, 11]. Interestingly, none of the PEs identified in this study concerned these drugs or drug classes.

Several classes of drugs for which PEs were made and identified in current study are not considered high-risk medications [32]. In total, 20.4% of PEs concerned PPIs (ATC-code A02B). These drugs were often prescribed without an appropriate medical indication. Deprescribing [33] should be attempted to avoid long-term inappropriate use, which can give rise to *Clostridium difficile* infections [34–37] or increased fracture risk [38, 39]. The prescription of vitamins, for example colecalciferol (ATC code A11CC05), was also associated with PEs. In this case, the lack of prescription of colecalciferol could lead to suboptimal treatment of osteoporosis and fracture risk [40]. Other drug classes associated with PEs were agents acting on the renin-angiotensin system (ATC-code C09) and antithrombotic agents (ATC-code B01). These drugs were previously considered high-risk medications [32], for example, causing hypotension resulting in collapse, and bleeding, or thromboembolic events. However, it should be borne in mind that, depending on patient characteristics and time, all PEs have the ability to cause patient harm. Therefore, timely identification and prevention of medication-related harm is crucial.

This study shows that the majority of the PEs identified were made during COVID-19 hospitalization. A PCOC visit provides the opportunity to identify PEs, thereby potentially reducing the risk of medication-related harm, emergency department visits, or (re)hospitalization [41]. Ideally, the medication of all patients should be reviewed for appropriateness, which would circumvent these problems and prevent the transmural transfer of PEs [42, 43]. We showed that an ICU admission during hospitalization and a medical history of COPD / asthma are independent risk factors for PEs. These findings can be used to identify in high-risk patients and implement targeted interventions.

That ICU admission was an independent risk factor for PEs is consistent with findings from before the pandemic [13]. ICU admission involves the transfer of care, which introduces the risk of incomplete transfer of information, resulting in changes in prescribed medication [13, 44–47]. It often necessitates the deliberate (temporary) discontinuation of (chronic) medication, resulting in a risk of medication omission at ICU discharge. Moreover, medication is often prescribed for ICU-specific indications, such as edema, infections, delirium, and cardiac disorders, often for temporary use [45–47]. Patients admitted to the ICU are prescribed twice the amount of medication prescribed to patients not admitted to the ICU [48]. Studies have shown that the absolute number of medications is a risk for medication-related harm [49, 50].

An additional problem is that, during the pandemic, the sharp increase in patients resulted in understaffing. To meet the demand, healthcare professionals from various specialties joined the frontline [4] and were expected to prescribe and decide over pharmacotherapeutic therapy beyond their own medical expertise. Inadequate and incomplete transfer of information on medication therapy would make it difficult hamper the identification of PEs.

In the absence of medication reconciliation [25], there was often inadequate information on the medications used before hospitalization. Medical treatment of COPD / asthma often involves at least one inhalational drug. In the admission letters analyzed, the name of the drug was replaced by the drug class (e.g. ‘inhalation medication’) or drug form (‘inhalator’) without specification of the dosage or dosage frequency. This probably explains why a medical history of COPD or asthma was a significant risk factor for PEs in COVID-19 patients.

Several studies, carried out before the pandemic, have focused on medication safety, but these used different definitions of PEs, which makes comparison difficult. The study of O’Riordan et al. [51] used the same definition for PEs [23] as we used and had a similar study design and method. These authors identified post-discharge PEs in 36 of 83 included patients, with unintentional drug omissions being the most common type of PE. In contrast, we found a much higher proportion of patients with PEs, with unintentional initiation of medication being the most common type of PE. The latter was possibly due to medication intended for temporary use, for example during ICU admission, not being discontinued at discharge.

We found that although patients who visited the PCOC were prescribed relatively few medicines, there were still almost 1.5 PE per patient identified. An earlier study from our group, performed before the pandemic, found a higher number of drugs prescribed per patient but an almost equal number of PEs per patient [12]. Thus our current findings appear to contradict an earlier observation that the number of prescribed drugs is associated with medication-related patient harm due to PEs [49].

Our findings also support the idea that the circumstances surrounding in-hospital prescribing changed during the pandemic, such that risk factors for PEs identified before the pandemic might not be applicable for risk stratification during a pandemic.

### Limitations and strengths

We believe this study has some major strengths. Firstly, to the best of our knowledge, this study is the first to evaluate the quality of pharmacotherapeutic care provided during the SARS-CoV-2 pandemic. Secondly, almost all patients scheduled for follow-up at the PCOC were included in this analysis. Thus results provide a realistic representation of potentially avoidable medication-related harm in times of a pandemic. Lastly, evaluation of the inappropriate prescriptions and discrepancies in a multidisciplinary consensus meeting provides a more balanced interpretation of the prevalence and severity of PEs and insight into in-hospital prescribing during a pandemic.

This study had some limitations. Firstly, the medication review was performed 3 months after hospital discharge. At that moment, assessment was thus dependent on the written communication available. If there was insufficient contextual information, someof the discrepancies identified may have been classified as ‘intentional’ and not identified as a PE. This means that the actual number of PEs in this cohort may have been higher than we reported. Secondly, our analysis included only patients who required follow-up and who came to the PCOC. Thus not all post-COVID-19 patients were included in this analysis, such as patients without complications during COVID-19-related hospitalization, patients not attending their appointment, and those who died before the PCOC visit. Therefore, the results of this study are not generalizable to all (post -)COVID-19 patients. Future research should involve all (post-)COVID-19 patients to determine the effectiveness of the suggested risk stratification.

## Conclusion

More than 90% of the post-COVID-19 patients in this study had ≥1 PE, 3 months after discharge and more than 8% required an immediate change in medication therapy (intervention) because of harm. ICU admission and a medical history of COPD or asthma were identified as independent risk factors for PEs. This is the first comprehensive investigation of PEs during the SARS-CoV-2 pandemic. PEs are an unanticipated challenge during a pandemic and can put extra pressure on already overstretched hospital services, especially when these errors give rise to patient harm. Further research should focus on interventions to prevent and reduce PEs. For example, whether a pharmacotherapeutic stewardship team, which could use the suggested risk stratification to identify high risk patients; review medication and; suggest modifications before hospital discharge, can reduce PEs and subsequent patient harm.

## Supplementary Information

**Additional file 1.**

**Additional file 2.**

**Additional file 3.**

**Additional file 4.**

**Additional file 5.**

**Additional file 6.**

**Additional file 7.**

## Data Availability

The data that support the findings of this study are available from the corresponding author upon reasonable request.
