# Supplementary figures and images for: Prescribing errors in post - COVID-19 patients: prevalence, severity, and risk factors in patients visiting a post - COVID-19 outpatient clinic
Source: BMC Emerg Med. 2022 Mar 5;22:35. doi: 10.1186/s12873-022-00588-7 (PMC8897739; doi:10.1186/s12873-022-00588-7)

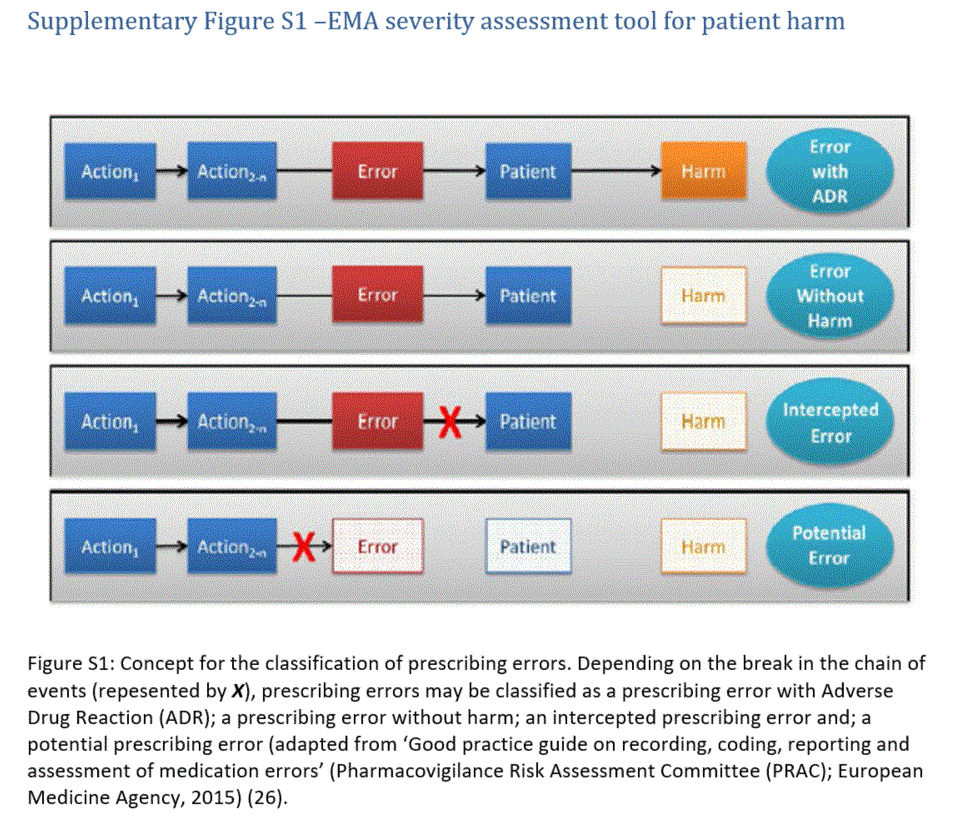

Supplement: Supplementary file 1 — Additional file 1. [file 12873_2022_588_MOESM1_ESM.gif]
