# Supplementary material for: Prescribing errors in post - COVID-19 patients: prevalence, severity, and risk factors in patients visiting a post - COVID-19 outpatient clinic
Source: BMC Emerg Med. 2022 Mar 5;22:35. doi: 10.1186/s12873-022-00588-7 (PMC8897739; doi:10.1186/s12873-022-00588-7)
Supplement: Supplementary file 3 — Additional file 3. [file 12873_2022_588_MOESM3_ESM.docx]

# Supplementary table S2 – documents medication analyses Post-COVID-19 Outpatient Clinic

## Table 1: General information

|  | **Date (dd-mm-yyyy)** | **Highlight** |
| --- | --- | --- |
| Is medication anamnesis performed prior to PCOC visit? | - | YES / NO |
| Physical consult with Pharmacotherapy team at PCOC necessary | - | YES / NO |
| Date of scheduled PCOC appointment |  | *-* |
| Did the patient show at PCOC appointment? | - | YES / NO |

## Table 2: Patient demographics

| **Patient information** | |
| --- | --- |
| Gender |  |
| Age at time of COVID-19 – related hospital admission |  |
| **Medical information** | |
| Pregnant / breastfeeding / child wish |  |
| Weight at time of COVID-19 – related hospital admission, in case known |  |
| Length at time of COVID-19 – related hospital admission, in case known |  |
| Allergies |  |
| *Current intoxications:* YES / NO | |
| - In case YES: Smoker? YES / NO |  |
| - In case YES: Alcohol use? YES / NO (+ number of U) |  |
| - In case YES: Drugs? YES / NO |  |
| **Admission** | |
| Transfer from other hospital: YES / NO |  |
| - In case YES: specify which hospital |  |
| - In case YES: specify admission- and discharge date from other hospital |  |
| Locatie opname: VU |  |
| - Date admission at Amsterdam UMC – location VUmc |  |
| - Date discharge at Amsterdam UMC – location VUmc |  |
| - Directly admitted at ICU: YES/NO |  |
| - When is COVID-19 contracted: prior to hospitalization / during hospitalization (either in other hospital or at Amsterdam UMC – location VUmc) |  |
| Transfer to other hospital: YES / NO |  |
| - In case YES: specify which hospital |  |
| Number of intramural transfers in Amsterdam UMC – location VUmc |  |
| CORADS score of first CT scan ***(A)**** |  |
| CT score of first CT scan |  |
| Positive PCR for SARS-CoV-2: YES / NO |  |
| ICU admission during hospitalization at Amsterdam UMC – location VUmc: YES / NO |  |
| - In case YES: Date of ICU admission and discharge |  |
| - In case YES: Intubated at ICU: YES / NO |  |
| - In case YES: Renal replacement therapy at ICU ***(B)*****: YES / NO |  |
| Complications during hospitalization at Amsterdam UMC – location VUmc  *(Non, DVT, pulmonary embolism, myocardial infarction, atrial fibrillation, CVA, delirium, superinfection, pneumothorax, electrolyte irregulation* |  |
| Patient had a tube during hospitalization at Amsterdam UMC – location VUmc:  YES / NO |  |
| - In case YES: specify what tube was placed |  |
| Treatment restrictions during hospitalization at Amsterdam UMC – location VUmc:  NON / No ICU / No reanimation / No mechanic ventilation |  |
| *Relevant laboratory diagnostics during hospitalization* |  |
| - Kidney function at admission (kreat, eGFR) |  |
| - Lowest kidney function during hospitalization (kreat, eGFR) |  |
| - Kidney function at discharge (kreat, eGFR) |  |
| Has medication reconciliation according to protocol taken place at admission at Amsterdam UMC – location VUmc? |  |
| **After hospital discharge** | |
| ED presentation(s): YES / NO |  |
| - In case YES: specify which hospital |  |
| - In case YES: date of ED-presentation(s) |  |
| - In case YES: specify reasons of ED-presentation(s) |  |
| - In case YES: is patient hospital readmitted: in case YES, date of hospital readmission and -discharge |  |
| **Living situation** | |
| Living situation prior to COVID-19 - hospitalization:  *(at home without extra care, at home with family care, at home with professional care, temporarily rehabilitation center, nursing home)* |  |
| Living situation prior to COVID-19 - discharge:  *(at home without extra care, at home with family care, at home with professional care, temporarily rehabilitation center, nursing home)* |  |
| Living situation at PCOC visit:  *(at home without extra care, at home with family care, at home with professional care, temporarily rehabilitation center, nursing home)* |  |
| **Notes** | |
|  |  |

**** (A).*** *CT-scan mad at ED of Amsterdam UMC location VUmc or other hospital. In case no CT scan is made, fill in ‘not applicable’.*

***** (B).*** *Kidney replacement therapy: CVVH, HD or peritoneal dialysis*

## Tabel 3. Medication use

| **1. Medical history including year of diagnosis, including morbidities developed during COVID-19 episode** | **2. In case of transfer from other hospital: Medication in use at admission of that hospital (drug name, dosage, frequency and route of administration)**  **According to transfer letter** | **3. Medication in use at admission of Amsterdam UMC location VUmc (drug name, dosage, frequency and route of administration)**  **According to admission letter of Amsterdam UMC** | **4. Medication in use at admission of Amsterdam UMC location VUmc (drug name, dosage, frequency and route of administration)**  **According to community pharmacy records of patient** | **6. Medication in use at discharge from Amsterdam UMC location VUmc (drug name, dosage, frequency and route of administration)**  **According to discharge letter of Amsterdam UMC** | **7. Medication in use at discharge from Amsterdam UMC location VUmc (drug name, dosage, frequency and route of administration)**  **According to prescribed medication** | **9. Medication in use at time of post-COVID-19 outpatient clinic visit (drug name, dosage, frequency and route of administration)** |
| --- | --- | --- | --- | --- | --- | --- |
|  |  |  |  |  |  |  |
|  |  |  |  |  |  |  |
|  |  |  |  |  |  |  |
|  |  |  |  |  |  |  |
|  |  |  |  |  |  |  |
|  |  |  |  |  |  |  |
|  |  |  |  |  |  |  |

## Table 4. Medication Interview

| **Step 1. Medication reconciliation (table 3, column 8)** | |
| --- | --- |
|  | |
| **Step 2.** | |
| - ED presentation between discharge and PCOC visit / re-admission at other hospital than Amsterdam UMC location VUmc |  |
| - Adverse drug events / medication-related problems? |  |
| - Over the counter medication in use?   *(pain medication, vitamins, supplements)* |  |
| - Length |  |
| - Weight |  |
| - Allergies and specify symptoms |  |
| - How does patient administer medication?   *(self-intake, help from family care, professional care a, Baxter, pill box, help from nurse in case of living in nursing home)* |  |
| **Step 3. Should patient be scheduled for a consult with the Pharmacotherapy team?**  **In case, yes: specify** | |
| - YES / NO |  |
| - *Specify* |  |

## Table 5. Consensus list medication at admission; discharge and PCOC visit

| **Consensus list medication at admission (CMA)** | **Consensus list medication at discharge (CMD)** | **Consensus list medication at PCOC visit (CMP)** |
| --- | --- | --- |
|  |  |  |
|  |  |  |
|  |  |  |
|  |  |  |
|  |  |  |
|  |  |  |
|  |  |  |
|  |  |  |
|  |  |  |
|  |  |  |
|  |  |  |
|  |  |  |
|  |  |  |
|  |  |  |
|  |  |  |
|  |  |  |
