# Supplementary material for: Prescribing errors in post - COVID-19 patients: prevalence, severity, and risk factors in patients visiting a post - COVID-19 outpatient clinic
Source: BMC Emerg Med. 2022 Mar 5;22:35. doi: 10.1186/s12873-022-00588-7 (PMC8897739; doi:10.1186/s12873-022-00588-7)
Supplement: Supplementary file 4 — Additional file 4. [file 12873_2022_588_MOESM4_ESM.docx]

# Supplemental table S3 – NCC MERP Index ^27^

|  | NCC MERP Category | Description |
| --- | --- | --- |
| No error | A | Circumstances or events that have the capacity to cause error |
| Error, no harm | B | An error occurred but did not reach the patient and thus is clinically not relevant.  *An error of omissions does reach the patient.* |
|  | C | An error occurred and reached the patient (is clinically relevant) but did not cause patient harm. |
|  | D | An error occurred that reached the patient (is clinically relevant) and needed monitoring to confirm it did not result in harm to the patient and/or required intervention to preclude patient harm. |
| Error, Harm | E | An error occurred that may have contributed to temporary patient harm and where intervention is required. |
|  | F | An error occurred that may have contributed to temporary patient harm and required initial or prolonged hospitalization. |
|  | G | An error occurred that may have contributed to or resulted in permanent patient harm. |
|  | H | An error occurred that required intervention to sustain life. |
| Error, Death | I | An error occurred that may have contributed to or resulted in patient’s dead. |

* Harm: Impairment of the physical, emotional or psychological function or structure of the body and/or pain resulting therefrom.

** Monitoring: To observe or record relevant physiological or psychological signs.

*** Intervention: May include change in therapy or active medical/surgical treatment

**** Intervention necessary to sustain life: Includes cardiovascular and respiratory support (e.g. CPR, defibrillation, intubation, etc.)
