# Supplementary material for: Prescribing errors in post - COVID-19 patients: prevalence, severity, and risk factors in patients visiting a post - COVID-19 outpatient clinic
Source: BMC Emerg Med. 2022 Mar 5;22:35. doi: 10.1186/s12873-022-00588-7 (PMC8897739; doi:10.1186/s12873-022-00588-7)
Supplement: Supplementary file 5 — Additional file 5. [file 12873_2022_588_MOESM5_ESM.docx]

#### **Table S4. Examples of prescribing errors causing patient harm or no harm according to the EMA classification and level of severity according to the NCC MERP index**

| **Type of Prescribing Errors (PEs)** | | **Case description of patient harm** | **Case description of no harm** |
| --- | --- | --- | --- |
| **Unintentional discrepancies** | | | |
| **Unintentional initiation of a drug** | | • During ICU-admission patient of 63 years was prescribed captopril 6.25 mg 3 dd due to hypertension. At hospital discharge from clinical ward, patient was rather hypotensive (RR 117/59). However this could be due to being sick and follow up for RR-control was communicated to the GP. Captopril was switched to enalapril once daily 5 mg.  At PCOC visit, enalapril once daily 5 mg was in use, however patient was feeling dizzy for some time, after discharge especially while standing up. In combination with a low blood pressure this was diagnosed as orthostatic hypotension by pulmonologist. At PCOC enalapril was stopped and again follow up for RR-control was transferred to the GP (*NCC MERP category E*). | • At ICU thiamine once daily was started due to refeeding. This was continued after ICU discharge, at clinical ward and prescribed at discharge. There was no documented indication for continuation of thiamine this long or after hospital discharge. The patient was using it as prescribed at PCOC visit and was showing no harmful consequences from thiamine suppletion.  • At ICU quetiapine 25 mg 2dd and oxazepam 20mg 4dd PRN was started as standard protocol. This was prescribed at hospital discharge. However, there was no documented indication for continuation. The patient was not using it at time of PCOC visit and was showing no harmful consequences.  • Timolol and latanoprost eye droplets were after hospital admission prescribed for unknown reasons. However patient’s ophthalmologist stated in patient’s record explicitly that these two should not be (re)prescribed anymore. From the patient record, not clinically significant effects were reported.  • At hospital admission patient was using mirabegron 50 mg once daily. During hospitalization it was concluded that patient did not have any benefit from the drug and was therefore discontinued. At hospital discharge, this drug was not prescribed. At PCOC visit patient was using it again on prescription of the GP. We assumed the information on discontinuation had not reached the GP and was therefore prescribed again. Patient showed no signs of ADEs (20) but still had benefit from using the drug. |
| **Unintentional omission of a drug** | | • At hospital admission patient was using a combination tablet of valsartan once daily 320 mg / amlodipine once daily 10 mg / hydrochlorothiazide once daily 25 mg.  At admission patient was only using amlodipine 1dd 10 mg and valsartan once daily 80 mg. Hydrochlorothiazide was discontinued without rationale and patient was experiencing hypertension (*NCC MERP category E*). | • Before hospital admission a patient was using trazodone once daily 100 mg AN. I.e. due to a language barrier this was not prescribed during hospitalization. At PCOC visit trazodone was not in use, however due to increased psychiatric symptoms new antipsychotics were initiated. Due to limited information during hospitalization we were unable to determine patient harm. |
| **Unintentional switch of a drug within the same ATC-group** | | • Patient was using citalopram once daily 20 mg. After hospitalization this was adjusted to escitalopram once daily 20 mg. This is equivalent twice the usual dosage patient was using. Patient experienced ADEs related to SSRI (*NCC MERP category E*). | *none* |
| **Unintentional dosage change** | | *none* | • At admission pantoprazole 40 mg once daily was in use. For unknown reasons but without therapeutic indication, dosage was increased to twice daily. This was continued after hospitalization.  • Beclometason (FOSTER) 100/6 mcg twice daily 2 inhalations was in use at admission. At discharge the dosage was prescribed as 200/6 mcg/dose aerosol twice daily 2 inhalations. There was no rationale documented for this dosage increase. |
| **Inappropriate medication use** | | | |
| **Core outcome** | **Specification** |  |  |
| **Underuse** | Incomplete pharmacotherapy according to relevant guideline or protocol | • During COVID-hospitalization isosorbidemononitraat once daily 50 mg MGA was in use for angina pectoris. At time of PCOC visit this was not prescribed anymore. As the rationale for discontinuation could not be detected but patient was still using short acting nitroglycerine spray PRN, this should be prescribed according to guidelines. There were no signs of ADEs (20) at time of PCOC (*NCC MERP category E*). | • A proton pump inhibitor (PPI) was not prescribed as prophylaxis for the gastrointestinal side effects of diclofenac 3dd 50 mg (NSAIDs**) for rheumatic pain in a patient of 72 years. Patient did not experienced ADEs (20) of NSAIDs**  • No preventive laxative prescribed during opioid use, without signs of diarrhoea. This did not result in opioid-related obstipation. |
|  | Incorrect duration (too short) of prescribed drug therapy | *none* | • Insufficient rivaroxaban once daily 20 mg was prescribed for COVID-19 – related pulmonary embolisms requiring 3 months of treatment. Patient stopped therapy preliminary. No signs of ADEs (20) or pulmonary embolisms were seens at PCOC visit. |
| **Overuse** | Drug continued despite no indication (anymore) (*desprescribing*) | *none* | • A proton pump inhibitor (PPI) was prescribed as prophylaxis for the gastrointestinal side effects of NSAIDs**. During admission the NSAID** was discontinuated, however the PPI was not and had no other indication than as prophylaxis of NSAIDs**.  • Between admission and discharge atorvastatin once daily 40 mg was prescribed parallel to ezetimibe 10 mg once daily. This was still parallel in use at time of PCOC visit. Documentation and patient claimed this combination was stopped in the weeks before COVID-hospitalization. |
|  | (Pseudo) drug duplication | *none* | • Omeprazole once daily 40 mg was in use at admission. During hospitalization pantoprazole once daily 40 mg was in addition prescribed and remained prescribed at discharge. The indication for use of omeprazole once daily 40 mg prior to COVID-related hospitalization was unknown. During hospitalization, double PPI-use remained unknown. At PCOC visit both PPIs were still parallel in use. There was no therapeutic indication or prophylactic indication for a PPI based on patient’s record or medication anamnesis. Patient however showed no signs of ADEs (20). |
|  | No, unknown or incorrect indication of a drug | *none* | • Patient was prescribed folic acid once daily 5 mg. There was no documented indication for use and patient was not unaware why this was prescribed and in use. |
| **-** | Incorrect dosing  *- Under- or overdosing*  *- Incorrect dosing frequency* | • At admission metformin twice daily 1000 mg was in use next to gliclazid once daily retard 120 mg and 1dd NOVOMIX 30/70 E/ml; 20IE in the morning and 10IE in the afternoon for diabetes mellitus type 2. During hospitalization metformin was adjusted to once daily 1000 mg due to low blood glucose levels and NOVOMIX 30/70 E/ml was suspended. Gliclazid remained unchanged. At discharge no changes in diabetes medication was not adequately communicated to the GP and the regimen as described remained the same.  At PCOC patient’s blood glucose levels (fasting) was above 20 mmol/L while metformin was still used once daily 1000 mg, gliclazid remained unchanged and NOVOMIX 30/70 E/ml was prescribed at 2 dd 6 EH (*NCC MERP category E*). | • Metoprolol tartrate 3dd 50 mg was prescribed during ICU-admission for AF de novo. At discharge this was continued and at PCOC visit still at use. According to guidelines, metoprolol tartrate for AF should be dosed twice daily. Switch to metoprolol succinate should be considered. No signs of ADEs or inconveniences were reported.  • Rivaroxaban 15 mg once daily was prescribed for atrial fibrillation de novo with a CHADVASC = 4. However there were no signs of reduced kidney function so 20 mg once daily was in place. There were no signs of ADEs or inconveniences were reported. |
| **Potentially inappropriate medications** | Drug should be discontinued due to an adverse drug event (ADE), intoleration or contra-indication | • Amitriptyline 10 mg was in use for restless legs syndrome. Patient indicated signs similar due to ADEs of amitriptyline, like signs of anticholinergic syndrome, in combination with age (68 years) an equivalent dose of nortriptyline was more appropriate (*NCC MERP category E*). | • While an ACE-inhibitor was more appropriate after myocardial infarction, a calcium antagonist was in use (amlodipine once daily 10 mg). Due to edema, furosemide once daily 40 mg was initiated. No other indication for furosemide was discovered. As edema is a common (> 10%) ADE of calcium antagonists, furosemide was suspected to be prescribed for an ADE of amlodipine, while an ACE-inhibitor or angiotensin-inhibitor was more in place according to guidelines and perhaps furosemide can be re-evaluated. |

* PRN, pro re nata = ‘when necessary’.

** NSAIDs, non-steroidal anti-inflammatory drugs

*** AN, Ante Noctum = ‘for the night’
