# Supplementary material for: Prescribing errors in post - COVID-19 patients: prevalence, severity, and risk factors in patients visiting a post - COVID-19 outpatient clinic
Source: BMC Emerg Med. 2022 Mar 5;22:35. doi: 10.1186/s12873-022-00588-7 (PMC8897739; doi:10.1186/s12873-022-00588-7)
Supplement: Supplementary file 6 — Additional file 6. [file 12873_2022_588_MOESM6_ESM.docx]

# Supplementary table S5 - Number of identified prescribing errors per Anatomical Therapeutic Chemical code

|  | **ATC code** | **Drug categories based on ATC code** | **Number of PEs (%)** |
| --- | --- | --- | --- |
| **1.** | A02 | **DRUGS FOR ACID RELATED DISORDERS** | 30 (20.4) |
| **2.** | A11 | **VITAMINS** | 24 (16.3) |
| **3.** | C09 | **AGENTS ACTING ON THE RENIN-ANGIOTENSIN SYSTEM** | 12 (8.2) |
| **4.** | B01 | **ANTITHROMBOTIC AGENTS** | 10 (6.8) |
| **5.** | N02 | **ANALGESICS** | 9 (6.1) |
