# Supplementary material for: Prescribing errors in post - COVID-19 patients: prevalence, severity, and risk factors in patients visiting a post - COVID-19 outpatient clinic
Source: BMC Emerg Med. 2022 Mar 5;22:35. doi: 10.1186/s12873-022-00588-7 (PMC8897739; doi:10.1186/s12873-022-00588-7)
Supplement: Supplementary file 7 — Additional file 7. [file 12873_2022_588_MOESM7_ESM.docx]

# Supplementary table S6 A - UNIVARIATE ANALYSES: Combined ≥1 unintentional drug discrepancy medication at admission vs medication at discharge and a PE introduced during hospitalization

| **Variable** | **Group** |  |  |  |  |
| --- | --- | --- | --- | --- | --- |
|  |  | **p-value** | **OR*** | **95% CI** | |
| Gender | Male | 0,94 | 0,96 | 0,36 | 2,54 |
|  | Female |  |  |  |  |
| Living situation prior to COVID-19 - hospitalization - at home without professional care | No | **0,00** | 8,90 | 1,60 | 49,20 |
|  | Yes |  |  |  |  |
| Transferred from another hospital to Amsterdam UMC location VUmc | No | 0,11 | 2,20 | 0,84 | 5,77 |
|  | Yes |  |  |  |  |
| Admitted directly at ICU at admission at admission at Amsterdam UMC - location VUmc | No | **0,00** | 5,51 | 2,03 | 14,96 |
|  | Yes |  |  |  |  |
| Smoker | No | 0,12 | 3,63 | 0,67 | 19,71 |
|  | Yes |  |  |  |  |
| Comorbidity Hypertension | No | 0,36 | 1,55 | 0,60 | 3,97 |
|  | Yes |  |  |  |  |
| Comorbidity Diabetes Mellitus | No | 0,07 | 2,77 | 0,90 | 8,46 |
|  | Yes |  |  |  |  |
| Comorbidity Coronary Artery Diseases | No | 0,07 | - |  |  |
|  | Yes |  |  |  |  |
| Comorbidity Asthma / COPD | No | **0,01** | 5,29 | 1,50 | 18,64 |
|  | Yes |  |  |  |  |
| ICU admission during hospitalization at Amsterdam UMC - location VUmc | No | **0,00** | 6,04 | 2,24 | 16,25 |
|  | Yes |  |  |  |  |
| ICU admission from clinical ward of Amsterdam UMC - location VUmc | No | 0,38 | 1,80 | 0,48 | 6,74 |
|  | Yes |  |  |  |  |
| Intubated at ICU admission | No | **0,00** | 4,92 | 1,86 | 13,02 |
|  | Yes |  |  |  |  |
| Renal replacement therapy at ICU admission | No | 0,23 | - |  |  |
|  | Yes |  |  |  |  |
| Developed pulmonary embolisms during hospitalization at Amsterdam UMC - location VUmc | No | **0,01** | 4,00 | 1,37 | 11,71 |
|  | Yes |  |  |  |  |
| Developed delirium during hospitalization at Amsterdam UMC - location VUmc | No | **0,01** | 3,39 | 1,23 | 9,32 |
|  | Yes |  |  |  |  |
| Proven infection other than SARS-CoV-2 during hospitalization at Amsterdam UMC - location VUmc | No | **0,02** | 4,29 | 1,18 | 15,62 |
|  | Yes |  |  |  |  |
| Medication reconciliation at admission performed accordingly to the standard of care at Amsterdam UMC - location VUmc | No | 0,11 | - |  |  |
|  | Yes |  |  |  |  |
| Discrepancies (both intentional as unintentional) in admission letter of the first hospital patient was admitted to | No | **0,04** | 2,92 | 1,05 | 8,14 |
|  | Yes |  |  |  |  |
| Discrepancies (both intentional as unintentional) in discharge letter at Amsterdam UMC - location VUmc | No | 0,73 | 1,18 | 0,47 | 2,92 |
|  | Yes |  |  |  |  |
|  | |  |  |  |  |
| Age in years | | 0,48 | 1,01 | 0,98 | 1,05 |
| BMI | | 0,98 | 1,00 | 0,89 | 1,11 |
| CT-score at admission | | 0,97 | 1,00 | 0,90 | 1,12 |
| Total Charlson Comorbidity Index score | | 0,33 | 1,31 | 0,76 | 2,28 |
| Length of hospital admission in days | | **0,00** | 1,05 | 1,02 | 1,09 |
| The number of intramural transfers | | 0,14 | 1,33 | 0,91 | 1,95 |
| Length of ICU admission in days | | **0,01** | 1,06 | 1,02 | 1,11 |
| Number of unique drugs in CMA | | 0,09 | 1,10 | 0,99 | 1,23 |

# Supplementary table S6 B. UNIVARIATE ANALYSES: Combined ≥1 unintentional drug discrepancy medication at discharge vs medication at PCOC and a PE introduced after hospitalization

| **Variable** | **Group** |  |  |  |  |
| --- | --- | --- | --- | --- | --- |
|  |  | **p-value** | **OR*** | **95% CI** | |
| Gender | Male | 0,91 | 0,94 | 0,32 | 2,76 |
|  | Female |  |  |  |  |
| Living situation prior to COVID-19 - hospitalization - at home without professional care | No | 0,52 | 1,70 | 0,31 | 9,70 |
|  | Yes |  |  |  |  |
| Transferred from another hospital to Amsterdam UMC - location VUmc | No | 0,66 | 1,27 | 0,43 | 3,79 |
|  | Yes |  |  |  |  |
| Admitted directly at ICU at admission | No | **0,00** | 4,74 | 1,64 | 13,71 |
|  | Yes |  |  |  |  |
| Smoker | No | 0,29 | 2,58 | 0,42 | 15,73 |
|  | Yes |  |  |  |  |
| Comorbidity Hypertension | No | **0,00** | 6,84 | 2,29 | 20,48 |
|  | Yes |  |  |  |  |
| Comorbidity Diabetes Mellitus | No | 0,19 | 2,21 | 0,66 | 7,36 |
|  | Yes |  |  |  |  |
| Comorbidity Coronary Artery Diseases | No | 0,51 | 0,49 | 0,06 | 4,20 |
|  | Yes |  |  |  |  |
| Comorbidity Asthma / COPD | No | 0,60 | 1,46 | 0,35 | 6,00 |
|  | Yes |  |  |  |  |
| ICU admission during hospitalization at Amsterdam UMC - location VUmc | No | 0,11 | 2,26 | 0,82 | 6,25 |
|  | Yes |  |  |  |  |
| ICU admission from clinical ward of Amsterdam UMC - location VUmc | No | 0,08 | - |  |  |
|  | Yes |  |  |  |  |
| Intubated at ICU admission | No | 0,18 | 2,01 | 0,71 | 5,67 |
|  | Yes |  |  |  |  |
| Renal replacement therapy at ICU admission | No | 0,11 | 4,53 | 0,60 | 34,46 |
|  | Yes |  |  |  |  |
| Developed pulmonary embolisms during hospitalization at Amsterdam UMC - location VUmc | No | 0,75 | 0,80 | 0,21 | 3,10 |
|  | Yes |  |  |  |  |
| Developed delirium during hospitalization at Amsterdam UMC - location VUmc | No | **0,02** | 3,38 | 1,15 | 9,93 |
|  | Yes |  |  |  |  |
| Proven infection other than SARS-CoV-2 during hospitalization at Amsterdam UMC - location VUmc | No | 0,91 | 0,92 | 0,18 | 4,63 |
|  | Yes |  |  |  |  |
| Medication reconciliation at admission performed accordingly to the standard of care at Amsterdam UMC - location VUmc | No | 0,72 | 0,68 | 0,08 | 5,97 |
|  | Yes |  |  |  |  |
| Independent intake of medication at PCOC visit | No | 0,60 | 0,69 | 0,17 | 2,82 |
|  | Yes |  |  |  |  |
| Emergency department visit between hospital discharge and PCOC visit | No | 0,10 | 2,58 | 0,82 | 8,11 |
|  | Yes |  |  |  |  |
| Emergency department visit was related to COVID-19 | No | 0,72 | 0,68 | 0,08 | 5,97 |
|  | Yes |  |  |  |  |
| Emergency department visit resulting in hospital (re)admission | No | 0,86 | 0,82 | 0,09 | 7,48 |
|  | Yes |  |  |  |  |
| Discrepancies (both intentional as unintentional) in admission letter of the first hospital patient was admitted to | No | **0,04** | 3,30 | 1,01 | 10,84 |
|  | Yes |  |  |  |  |
| Discrepancies (both intentional as unintentional) in discharge letter at Amsterdam UMC - location VUmc | No | 0,39 | 1,56 | 0,57 | 4,30 |
|  | Yes |  |  |  |  |
| ≥1 unintentional medication discrepancy between admission and discharge | No | **0,00** | 5,16 | 1,77 | 15,04 |
|  | Yes |  |  |  |  |

|  |  |  |  |  |
| --- | --- | --- | --- | --- |
| Age in years | 0,17 | 1,03 | 0,99 | 1,07 |
| BMI | 0,65 | 1,03 | 0,91 | 1,16 |
| CT-score at admission | 0,76 | 1,02 | 0,90 | 1,16 |
| Total Charlson Comorbidity Index score | 0,76 | 0,90 | 0,46 | 1,78 |
| Length of hospital admission in days | **0,03** | 1,04 | 1,00 | 1,07 |
| The number of intramural transfers | 0,96 | 1,01 | 0,66 | 1,55 |
| Length of ICU admission in days | 0,17 | 1,03 | 0,99 | 1,07 |
| Number of unique drugs in CMA | **0,00** | 1,23 | 1,08 | 1,39 |
| Number of days between hospital discharge and PCOC visit | **0,04** | 0,98 | 0,95 | 1,00 |
| Number of unique drugs in CMD | **0,00** | 1,29 | 1,12 | 1,48 |

# Supplementary table S6 C. MULTIVARIATE ANALYSIS

| **Variable** | **p-value** | **OR*** | **95% CI** | |
| --- | --- | --- | --- | --- |
| ICU admission during hospitalization at Amsterdam UMC - location VUmc | **0,00** | 6,08 | 2,16 | 17,09 |
| Comorbidity Asthma / COPD | **0,02** | 5,36 | 1,34 | 21,50 |
